# Factors Associated With Candidiasis in Systemic Lupus Erythematosus Patients in Cipto Mangunkusumo National General Hospital: A Single-Center Case-Control Study

**DOI:** 10.7759/cureus.27107

**Published:** 2022-07-21

**Authors:** Ivan Damara, Kevin Winston, Frisky Maulida, Anna Ariane

**Affiliations:** 1 Internal Medicine, Cipto Mangunkusumo National General Hospital, Faculty of Medicine, Universitas Indonesia, Jakarta, IDN; 2 Hospital Medicine, Bhakti Medicare Hospital, Sukabumi, IDN; 3 Rheumatology, Cipto Mangunkusumo National General Hospital, Faculty of Medicine, Universitas Indonesia, Jakarta, IDN

**Keywords:** systemic lupus erythematosus, internal medicine, lymphocyte, corticosteroid, lupus, candidiasis

## Abstract

Background: Infection has been a major cause of morbidity and mortality in systemic lupus erythematosus (SLE) patients. One of the infections encountered in SLE patients is candidiasis. Therefore, we aimed to conduct a case-control study to explore the risk factors associated with candidiasis in SLE patients in our center.

Methods: Medical records of 20 SLE patients with fungal infection were obtained, and a case-control study was conducted with an age and sex-matched control group of 20 patients. Data were obtained from the Cipto Mangunkusumo National General Hospital. SLE confirmatory diagnosis was based on Systemic Lupus Erythematosus International Collaborating Clinics (SLICC) 2012 criteria. Patients with comorbidities of various chronic diseases (diabetes, HIV, and chronic kidney disease) were excluded. Statistical analysis was conducted using the Mann-Whitney U test with statistical significance defined as a p-value < 0.05.

Result: Based on the analysis, a maximum corticosteroid dose of 24 (4-250) mg/day for the last one year was associated with the development of fungal infection (p = 0.047). Lower absolute lymphocyte count (748 cells/mm³ versus 1635 cells/mm³) was also associated with the occurrence of candidiasis in SLE patients (p = 0.008).

Conclusion: Physicians should be aware that corticosteroid use in SLE patients may cause candidiasis. Monitoring of maximum corticosteroid dose and absolute lymphocyte count is important to help prevent candidiasis. Patients should also be educated regarding the risk of candidiasis from corticosteroid use.

## Introduction

Systemic lupus erythematosus (SLE) is a chronic autoimmune disease involving multiple organ systems. The disease most commonly affects the skin, joints, vessels, serous membranes, heart, and central nervous system [1-3]. With its diverse manifestations from mild cutaneous and articular involvement to fatigue, cognitive impairment, and end-stage renal disease, SLE has the potential to cause substantial physical and functional disability [1,4].

The global burden of SLE is 6-35 new cases per 100,000 per year, with up to 90% of cases occurring in women of reproductive age. It has been reported that both the incidence and prevalence of the disease among Asian people are two to three times higher than in the white population [3]. Although some countries in Asia show a higher or lower prevalence of SLE, most studies show clustering prevalence values between 30 and 50 cases per 100,000 [5]. Meanwhile, candidiasis in SLE reaches up to 3% of total cases of SLE in several studies [6,7]. Economically, the disease may cause direct costs that include healthcare and non-healthcare costs, and indirect costs such as lost productivity due to disabilities the disease caused to the patient [4].

Infection is a major cause of morbidity and mortality in SLE patients [6]. Candidiasis accounts for the third most common infection in SLE patients after bacterial and viral infections. These infections commonly affect respiratory airways, urinary tract, soft tissue, and skin. *Candida* is known to be the most common opportunistic candidiasis, notably in the oral cavity. Even though oral infection is not fatal, it has been shown that disseminated *Candida* infection can originate from the oral cavity [8].

Commonly used drugs such as corticosteroid and immunosuppressive agents have been used in the early stage of SLE to prevent or minimize major organ damage, but these drugs also present with potential side effects that include infections [9]. With their anti-inflammatory activity, corticosteroids have been associated with infection susceptibility due to interference with T-lymphocyte-mediated immunity, monocyte, and macrophage system, as well as endothelial cells. The risk of developing an infection is also related to the high dosage and duration of the treatment [3].

Studies of SLE patients have also shown that certain pharmacological treatments such as cytotoxic agents and CD4+ count are associated with candidiasis or infections [5]. There are many studies that state CD4-mediated cellular immunity is required to prevent infection from *Candida* [10-13]. In this research, we would like to analyze the association of maximum corticosteroid dose, latest corticosteroid dose, and absolute lymphocyte count with the development of candidiasis in SLE patients.

## Materials and methods

A case-control design was used for this study. The data used in this research were secondary data obtained from the medical records of SLE patients of the Cipto Mangunkusumo National General Hospital, Faculty of Medicine, Universitas Indonesia, Jakarta, Indonesia. The population of this research was SLE patients diagnosed with candidiasis in the year 2010 to 2017. Inclusion criteria were adult patients aged ≥ 18 years old, diagnosed with SLE according to Systemic Lupus Erythematosus International Collaborating Clinics (SLICC) 2012 criteria, and diagnosed with candidiasis based on microscope analysis and/or clinical diagnosis. Exclusion criteria were medical records with incomplete data and patients who had comorbidities such as HIV/AIDS, chronic heart failure, kidney disease, liver disease, and other autoimmune diseases. After obtaining medical records of patients according to the inclusion and exclusion criteria, the data of the corresponding age and sex-matched control group were then obtained.

The outcome studied in this case-control study was any form of candidiasis (skin, oral, oropharynx, esophagus, and vaginal). The independent variables studied were a maximal dose of corticosteroid in the last one year before candidiasis diagnosis, the latest dose of corticosteroid, a three-month cumulative dose of corticosteroid, and absolute lymphocyte count. If there were multiple laboratory data on absolute lymphocyte counts, the nearest absolute lymphocyte count to the candidiasis diagnosis was used. Absolute lymphocyte count was calculated using data on total leukocyte and lymphocyte percentage.

Statistical analysis

Data obtained from medical records were analyzed using SPSS for Windows version 20.0.0 (IBM Corp., Armonk, NY). There were two phases of analysis. The first phase was univariate analysis to describe the data of samples obtained and the second phase was bivariate analysis to analyze the association between the risk factors and candidiasis development.

Univariate analysis for numerical data was conducted by presenting data characteristics in the form of mean and standard deviation for data that were distributed normally, or median and range for data that were not distributed normally. Categorical data were presented using the total number and percentage. The normality of the data was tested using the Shapiro-Wilk test, as there were less than 50 patients in this study. Statistical analysis was conducted using an independent t-test or Mann-Whitney U test depending on data distribution normality. Statistical significance was defined as a p-value < 0.05.

Ethics

As this study was a case-control study, ethical approval was not required by our institution.

## Results

The data of the research subjects in the form of medical records were taken from the central medical record unit of Cipto Mangunkusumo National General Hospital. The medical records taken were from patients diagnosed with SLE who developed candidiasis during the course of the disease. Initially, the medical records of 56 patients were obtained. Thirty of them had insufficient data, while six others presented with diseases of exclusion criteria, resulting in a total of 20 medical records that were included as research subjects. Another 20 medical records were obtained to be the control group of this research, matching the age and sex of the initial 20 cases. Thus, a total of 40 patients consisting of 20 SLE patients with candidiasis and 20 SLE patients without candidiasis were obtained.

From the data of SLE patients with candidiasis collected from the medical records, most of the subjects were female (95%) with an average age of 36.25 ± 9.44 years. Characteristics of the subjects can be seen in Table 1. No data were normally distributed according to the Shapiro-Wilk test.

**Table 1:** Characteristics of systemic lupus erythematosus patients who developed candidiasis (n = 20)

Variables	N (%)
Age ≤ 35	10 (50%)
Female gender	19 (95%)
*Candida albicans* fungal species	18 (90%)
*Candida krusei* fungal species	1 (5%)
Unidentified *Candida* sp. fungal species	1 (5%)
Skin site of candidiasis infection	2 (10%)
Oral site of candidiasis infection	11 (55%)
Oropharynx site of candidiasis infection	2 (10%)
Esophagus site of candidiasis infection	1 (5%)
Vagina site of candidiasis infection	4 (20%)
Hydroxychloroquine consumption	7 (35%)
Azathioprine consumption	4 (20%)
Mycophenolate mofetil consumption	6 (30%)

Relationship between each risk factor and candidiasis

As seen in Table 2, from the variables that have been analyzed, only two risk factors had a significant association with the occurrence of candidiasis (p < 0.05). The first risk factor was a maximal dose of corticosteroid (p = 0.047). In SLE patients with candidiasis, the median maximal dose was 24 mg/day, ranging from 4 to 250 mg/day. In contrast, SLE patients with no candidiasis had median maximum doses of 8 mg/day, ranging from 2 to 125 mg/day.

**Table 2:** Bivariate analysis

Variables	SLE patients who developed candidiasis	SLE patients who did not developed candidiasis	P-value
Maximal dose of corticosteroid (mg/day)	24 (4-250)	8 (2-125)	0.047
Latest dose of corticosteroid (mg/day)	16 (4-48)	8 (2-48)	0.121
3 months cumulative dose of corticosteroid (mg)	1180 (360-4320)	720 (180-4320)	0.366
Absolute lymphocyte count (cells/mm³)	748 (99-3312)	1635 (259-2743)	0.008

The second significant risk factor in this study was lower absolute lymphocyte count (p = 0.008). In the group that developed candidiasis, the median lymphocyte count was 748 cells/mm³, ranging from 99 to 3312 cells/mm³. Meanwhile, the group that did not develop candidiasis had a median lymphocyte count of 1635 cells/mm³, ranging from 259 to 2743 cells/mm³.

The latest administered dose of corticosteroid had no significant influence on candidiasis (p = 0.121). The total amount of corticosteroid administered within the last three months was also not significantly associated with candidiasis (p = 0.366).

## Discussion

Candidiasis in SLE patients

Infection is a major cause of mortality and morbidity in SLE patients; however, the proportion of SLE patients who develop candidiasis appears to be rare. One retrospective study consisting of 2344 Taiwanese SLE patients during a 26-year period only reported 15 cases of candidiasis (0.64%) [5]. Another study recorded 10 cases of candidiasis in 309 patients admitted with SLE (3.2%) [6].

In immunocompetent patients, *Candida* frequently colonizes the oral cavity and gastrointestinal tract without developing clinical manifestations [8]. However, in SLE patients, there are several risk factors, including a lower salivary flow rate that is common among SLE patients. Even though oral candidiasis might not be considered dangerous or fatal, it may spread into disseminated *Candida* infection [8]. The most common factors predisposing candidiasis in SLE patients were the use of cytotoxic drug therapy, heroin addiction, surgery, cardiac prostheses, antibiotic use, hemolytic anemia, Systemic Lupus Erythematosus Disease Activity Index (SLEDAI) score > 7, and high dose (more than 1000 mg/month) of glucocorticoid [5].

Corticosteroid use in SLE patients with candidiasis

Corticosteroid is one of the main drugs for SLE treatment. The use of this drug to suppress SLE disease activity also causes downstream effects, such as T-cell depletion, due to the inhibition of all cytokine transcription [14]. This condition, in turn, has been linked with susceptibility to candidiasis. In this study, several aspects of corticosteroid use have been recorded, i.e., latest dose, maximum dose, and three-month cumulative dose. However, the only statistically significant result was the amount of maximum dose administered before the development of candidiasis (p = 0.047). The statistical insignificance of both the latest dose and cumulative dose of corticosteroid in candidiasis development may be due to incomplete data or the small sample size of this study.

This study also agrees with previous studies that suggest the use of high-dose corticosteroids is a predisposing factor to candidiasis [8,15,16]. There are several mechanisms proposed for how corticosteroid use can lead to candidiasis. A study by Chen et al. consisting of 11 SLE patients with invasive fungal infection showed that maximum prednisolone exposure ≥ 45 mg/day was associated with invasive fungal infection. There are several mechanisms proposed for how corticosteroid use can lead to candidiasis [15]. Corticosteroids work by binding to intracellular glucocorticoid receptors and inhibiting cytokine production, causing inhibition of immune function. T-cell depletion, for example, is most commonly linked to candidiasis in HIV patients and is decreased by inhibiting interleukin-2, blocking the Th1 differentiation, and promoting its apoptosis [14]. Other factors that may increase the risk of having an infection include the inhibition of other immune cells such as macrophages and B cells.

The immune system in correlation with candidiasis in SLE

Candidiasis is strongly linked to patients with immunodeficiency. HIV patients are one of those most commonly affected [17]. A low CD4 count is associated with candidiasis. In this study, absolute lymphocyte count was used as an approximation of CD4 count since CD4 count is not a routine lab exam in SLE patients, and also due to a study that says corticosteroid does not significantly inhibit B cells function and production [14]. According to Shapiro et al., absolute lymphocyte count as a surrogate marker of C4 has adequate sensitivities and specificities [18]. Thus, absolute lymphocyte count can be used in resource-limited settings.

In this study, it is shown that patients with candidiasis have lower absolute lymphocyte count compared to the control group. This is in line with other studies that showed low CD4 was associated with candidiasis [10-13]. Other components of the immune system, such as neutrophils and macrophages, are also inhibited by the use of corticosteroids and may increase the risk of infection in SLE patients [14]. However, these factors are not recorded in this study due to unavailable data on the medical record.

Study limitations

The main limitation of this study is the small sample size due to the low prevalence of candidiasis infection in SLE patients. Another limitation is that statistical power was not calculated prior to the study. Furthermore, this was a single-center, case-control study; hence, there may be bias in this study that affected the results. Additional studies are needed to confirm the results of this study, especially multi-center studies and studies with a larger sample size.

## Conclusions

Physicians should be aware that corticosteroid use in SLE patients may cause candidiasis. Monitoring of maximum corticosteroid dose and absolute lymphocyte count is important to help prevent candidiasis. Patients should also be educated regarding the risk of candidiasis from corticosteroid use.
